# Histological Evaluation and Gene Expression Profiling of Autophagy-Related Genes for Cartilage of Young and Senescent Rats

**DOI:** 10.3390/ijms21228607

**Published:** 2020-11-15

**Authors:** Consuelo Arias, Nicolás Saavedra, Karla Leal, Bélgica Vásquez, Dulcineia S. P. Abdalla, Luis A. Salazar

**Affiliations:** 1 Center of Molecular Biology and Pharmacogenetics, Scientific and Technological Bioresource Nucleus, Universidad de La Frontera, Av. Francisco Salazar 01145, Temuco 4811230, Chile; consuelo.arias@gmail.com (C.A.); nicolas.saavedra@ufrontera.cl (N.S.); k.leal.villegas@gmail.com (K.L.); 2 Carrera de Kinesiología, Facultad de Ciencias de la Salud, Universidad Autónoma de Chile, Av. Alemania 1090, Temuco 4810101, Chile; 3 Facultad de Ciencias de la Salud, Universidad de Tarapacá, Av. General Velásquez 1775, Arica 1000007, Chile; belgica.vasquez@ufrontera.cl; 4 Department of Clinical and Toxicological Analyses, Faculty of Pharmaceutical Sciences, Universidade de São Paulo, Avenida Professor Lineu Prestes 580, São Paulo CEP 05508-000, SP, Brazil; dspa@usp.br

**Keywords:** autophagy, osteoarthritis, aging

## Abstract

Autophagy is a cellular mechanism that protects cells from stress by digesting non-functional cellular components. In the cartilage, chondrocytes depend on autophagy as a principal mechanism to maintain cellular homeostasis. This protective role diminishes prior to the structural damage that normally occurs during aging. Considering that aging is the main risk factor for osteoarthritis, evaluating the expression of genes associated with autophagy in senescent cartilage might allow for the identification of potential therapeutic targets for treatment. Thus, we studied two groups of young and senescent rats. A histological analysis of cartilage and gene expression quantification for autophagy-related genes were performed. In aged cartilage, morphological changes were observed, such as an increase in cartilage degeneration as measured by the modified Mankin score, a decrease in the number of chondrocytes and collagen II (Col2a1), and an increase in matrix metalloproteinase 13 (Mmp13). Moreover, 84 genes associated with autophagy were evaluated by a PCR array analysis, and 15 of them were found to be significantly decreased with aging. Furthermore, an in silico analysis based on by two different bioinformatics software tools revealed that several processes including cellular homeostasis, autophagosome assembly, and aging—as well as several biological pathways such as autophagy, insulin-like growth factor 1 (IGF-1) signaling, PI3K (phosphoinositide 3-kinase)/AKT (serine/threonine kinase) signaling, and mammalian target of rapamycin (mTOR) signaling—were enriched. In conclusion, the analysis identified some potential targets for osteoarthritis treatment that would allow for the development of new therapeutic strategies for this chronic disease.

## 1. Introduction

Autophagy is a cellular mechanism that protects the cells from stress-induced damage by digesting non-functional cellular components into double-membrane vesicles and delivering them to lysosomes for subsequent degradation and recycling, thus allowing for metabolism maintenance and the promotion of cell viability [1,2]. Autophagy is integrated into multiple signal transduction pathways that respond to the amount of available nutrients, energy balance, cytokines, and growth factors [3]. Three types of autophagy have been described, including macro, micro, and chaperone mediated autophagy, differing between them in relation to the mode of delivery of the degradation contents to the lysosome [4,5,6,7].

The protecting role of autophagy is especially important in articular cartilage, since chondrocytes have a low rate of proliferation and renewal [8], depending on autophagy as principal mechanism to maintain cellular homeostasis and remove dysfunctional organelles and macromolecules [9,10,11,12]. By observing the formation of autophagic vesicles in cartilage as a measure of autophagy activity, Caramés et al. showed a significant reduction in the basal autophagy in cartilage of aged mice, emphasizing that the alteration occurred before of structural damage related to aging [9,13,14]. Correspondingly, it has been shown that a reduction in autophagy levels can accelerate the aging process, while its stimulation may have a potent anti-aging effect [13,15,16,17]. 

Osteoarthritis (OA) is a degenerative, complex, and multifactorial joint disease [18,19] whose main risk of factor is aging [10,19,20,21,22]. Several alterations are observed in aged cartilage including a decrease in autophagy activation [8,10,23], which is correlated with cell death and OA [8,12]. Age-related changes in articular cartilage suggest an imbalance on mechanisms that maintain cell homeostasis such as autophagy, and its decline could be an early event in age-related diseases, triggering mechanical and structural tissue alterations with a subsequent death of chondrocytes. Thus, we aimed to evaluate the expression of genes related to the autophagy pathway in articular cartilage and its morphologic characteristics in young and senescent rats in order to identify potential therapeutic targets involved in chondrocyte viability and cartilage health.

## 2. Results

### 2.1. Histopathological Age-Related Changes of Articular Cartilage

**Figure 1:**
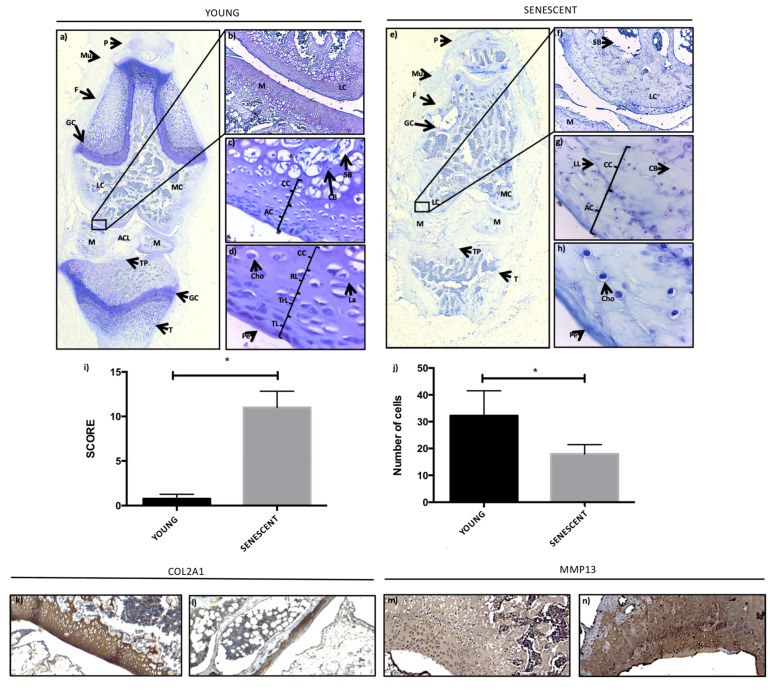
Histopathological comparisons of young and senescent rat knees: (**a**,**e**) Young and senescent rat knees, respectively. P: patella; Mu: muscle; F: femur; GC: growth cartilage; LC: lateral condyle; M: medial condyle; M: meniscus; ACL: anterior cruciate ligament; TP: tibial plateau; and T: Tibial. (**b**–**d**,**f**–**h**) Articular cartilage of young and senescent rat knees; respectively. Cho: chondrocytes; SB: spongy bone; CB: compact bone; AC: articular cartilage; CC: calcified cartilage; RL: radial layer; TrL: transitional layer; TL: tangential layer; and Pe: perichondrium. (**a**,**e**) The images were obtained with a Stereo Zoom Microscope Leica S6D 0.63×. (**b**,**f**) 15×; (**c**,**g**) 40×; (**d**,**h**) 100× and (**k**–**n**) 15× images were obtained with a Leica DM 2000 LED microscope and a digital camera (Leica MC 170 HD). (**i**) Modified Mankin Score. * *p* < 0.05. (**j**) Quantification of the number of chondrocytes in both groups, *p* < 0.05. (**k**–**m**) Immunohistochemistry of collagen II (Col2a1) and metalloproteinase 13 (Mmp13) in the young and senescent groups, respectively.

In the studied rat model, the senescent knee had some morphological differences compared to young knee (Figure 1). In the aged knee, a decreased staining was observed, suggesting a loss of extracellular matrix (Figure 1a,e). The young knee was characterized by a clear growth cartilage in femur and tibia, which was difficult to be observed in the aged ones (Figure 1a,e). The limiting line at the onset of the calcification zone of the cartilage was less demarcated in the aged knee group in comparison to the young knee group, probably because the areas close to the subchondral bone were not sufficiently calcified in the aged animals (Figure 1c,g); instead, there was a greater component of compact bone tissue in the meniscus, femur, and tibia in the old knee (Figure 1b,e,f). At a higher magnification in the young knee, we could visualize the chondrocytes whose morphology varied from flattened in the superficial areas to rounder in the deeper areas (Figure 1g). In some areas in the old knee, the different layers of the articular cartilage could not be determined, the superficial layers of cartilage had been lost, and the predominant form of chondrocytes was rounded (Figure 1h). Additionally, articular cartilage damage by aging was assessed by using the modified Mankin score. The young knees obtained a score of 0.75, and the senescent ones obtained a score of 11 from a total of 13 points. These differences were significant (Figure 1i). The empty lacunae and hypocellularity by age have already been described in several studies [7,20,23,24,25]; for this reason, we also quantified the number of cells. Consistently, in the old knee group, there was a significant decrease of the number of chondrocytes compared to the young knee group (Figure 1j).

Age-related changes impair the ability of chondrocytes to maintain the cartilage matrix. Thus, among the described alterations, a decrease in collagen II (Col2a1) by the action of matrix metalloproteinase 13 (Mmp13) has been observed [23,26,27]. Consistently, we observed a decreased Col2a1 expression and an increased Mmp13 expression in the old cartilage compared to the young cartilage after immunohistochemistry analysis.

### 2.2. Differential Expression of Genes Related to the Autophagy Pathway between Young and Senescent Groups

**Figure 2:**
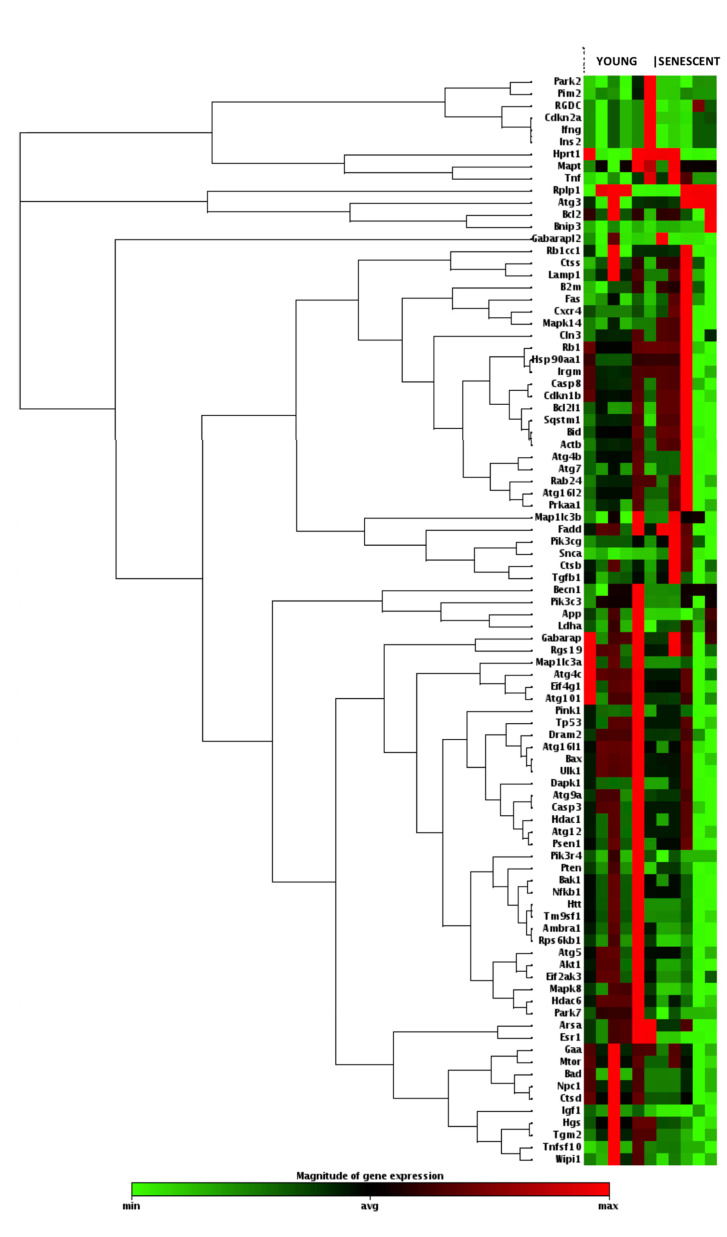
Heatmap of gene expressions and unsupervised hierarchical cluster analysis for genes of the autophagy pathway comparing young rats (3 weeks, N = 6) and senescent rats (25 months, N = 5). All data were normalized to the best housekeeping genes. Green = downregulated; red = upregulated. Magnitude of log2 (fold change): from −1.84 to 1.84.

**Figure 3:**
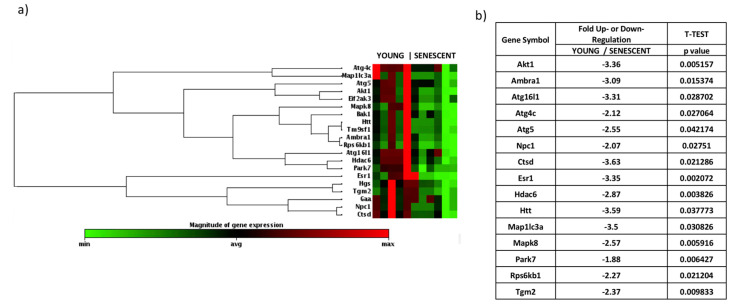
Differentially expressed genes of the autophagy pathway comparing the young knee and senescent knee groups. (**a**) Heatmap of gene expressions and unsupervised hierarchical cluster analysis for genes of the autophagy pathway comparing young rats (3 weeks, N = 6) and senescent rats (25 months, N = 5). The values are expressed in log2. All data were normalized to the best housekeeping genes. Green = downregulated; red = upregulated. Magnitude of log2 (fold change): from −1.84 to 1.84. (**b**) Fold changes of differentially expressed genes. Gene expression was evaluated using the ∆∆CT method, considering a fold change of >1.5 and Student’s t-test with a *p*-value < 0.05 as criteria. Negative values indicate downregulation, and positive values indicate upregulation. Akt1: V-akt murine thymoma viral oncogene homolog 1; Ambra1: Autophagy/beclin 1 regulator 1; Atg16l1: ATG16 autophagy related 16-like 1 (*S. cerevisiae*); Atg4c: ATG4 autophagy-related 4 homolog C (*S. cerevisiae*); Atg5: autophagy-related 5 homolog (*S. cerevisiae*); Npc1: Cdig2 protein; Ctsd: cathepsin D; Esr1: estrogen receptor 1; Hdac6: histone deacetylase 6; Htt: huntingtin; Map1lc3a: microtubule-associated protein 1 light chain 3 alpha; Mapk8: mitogen-activated protein kinase 8; Park7: Parkinson’s disease (autosomal recessive, early onset) 7; Rps6kb1: ribosomal protein S6 kinase, polypeptide 1; Tgm2: Transglutaminase 2, C polypeptide. *p*-values were calculated on the bases of Student’s t-test of replicate values of 2^ (-delta CT) for each gene for the senescent and young groups.

In order to identify differentially expressed genes related to the autophagy pathway in the cartilage of aged rats, a PCR array analysis was performed to compare young and senescent rats (RT^2^ Profiler Array Catalog N° PARN-084Z, Qiagen). Total RNA was isolated from cartilage and then reverse transcribed. The cDNA was used on the real-time RT^2^ Profiler PCR Array. CT values were normalized, and the data analysis web portal was used to calculate fold change/regulation by the delta-delta CT method. Fold change was calculated using the 2^ (-delta-delta CT) formula. This data analysis report was exported from the Qiagen web portal at GeneGlobe. Finally, a heatmap was obtained with a gene clustering dendrogram on the left, indicating linkage in the alteration profile (Figure 2). The PCR array included 84 autophagy-related genes, from which 15 presented significant differences between groups (Figure 3a,b). It is important to mention that all significantly deregulated genes in the senescent group had decreased expression levels. 

### 2.3. Bioinformatics Analysis

**Figure 4:**
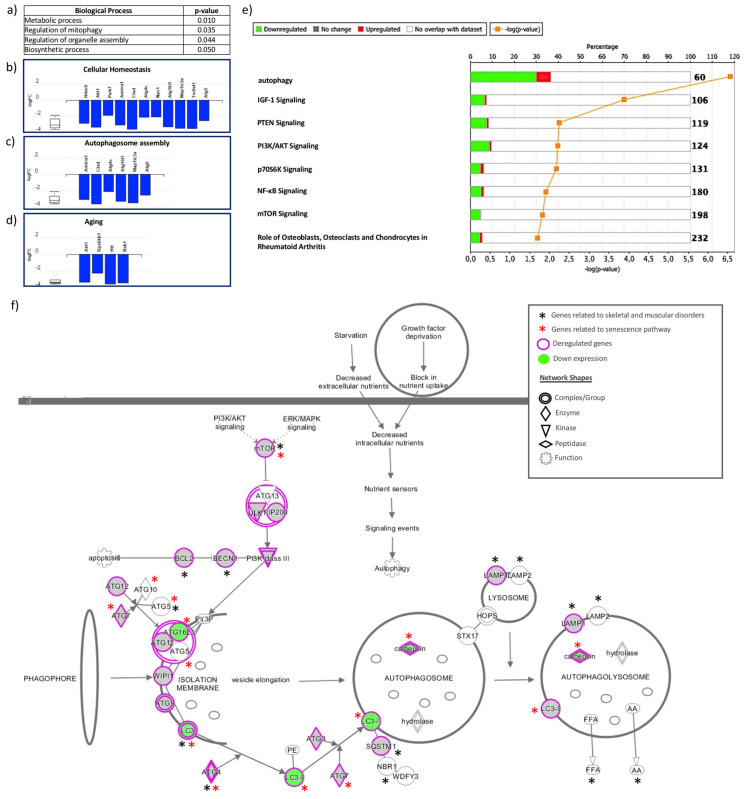
Bioinformatics analysis of biological process terms and pathways among genes related to autophagy in order to explore the biological relevance of the deregulated genes. (**a**,**d**) Bioinformatics analysis with Advaita Bio’s iPathwayGuide. (**a**) Table for biological process terms that are significantly associated with deregulated genes. (**b**–**d**) Molecules and their respective fold changes involved in the processes of cellular homeostasis, autophagosome assembly, and aging, respectively. (**e**,**f**) Bioinformatics analysis with Ingenuity Pathway Analysis. (**e**) Pathways and diseases associated with differentially expressed genes. (**f**) Autophagy pathway, deregulated genes, and overlap with skeletal and muscular disorder and senescence. One of the general ways to activate autophagy begins with growth factor deprivation that blocks the nutrient uptake, and another is starvation that decreases the extracellular nutrients; both stimuli decrease the intracellular nutrients, activate nutrient sensors, and generate signaling events that empower autophagy. The detailed processes are illustrated. The upstream pathways are PI3K (phosphoinositide 3-kinase)/AKT (serine/threonine kinase) signaling and ERK/MAPK signaling, respectively, which are able to regulate mammalian target of rapamycin (mTOR) signaling, a key factor for autophagy. In general, autophagy consists of a series of dynamic membrane rearrangements mediated by a group of proteins related to ATG, where Atg1 (ULK1), Atg6 (Beclin1), Atg8 (LC3), and Atg5 are the 4 major regulators of the autophagy pathway. First, cytoplasmic sequestration is generated within double membrane vesicles called autophagosomes. Subsequently, these vesicles are fused with the lysosome to generate autolysosomes, which leads to the degradation of the cargo. It is important to highlight the association among autophagy, skeletal and muscular disorders, and senescence. The genes involved in skeletal and muscular disorders are shown with the black asterisks, and the genes related to senescence are shown with red asterisks.

To identify potential associations between deregulated genes and cellular process, diseases, or functions, we performed a bioinformatics approach to explore the biological relevance of observed changes. We submitted a gene list showing significant differences between the analyzed groups to Advaita Bio’s iPathwayGuide (https://www.advaitabio.com/ipathwayguide) and to Ingenuity Pathway Analysis (IPA^®^, Qiagen, Redwood City, CA, USA). The Advaita Bio’s iPathwayGuide analysis linked our deregulation with processes such as cellular homeostasis and aging (Figure 4a,d). The IPA found a relationship with autophagy, insulin-like growth factor 1 (IGF-1) and PI3K (phosphoinositide 3-kinase)/AKT (serine/threonine kinase) signaling among others (Figure 4e). Furthermore, the IPA identified several genes that are involved in the autophagy pathway, the senescence pathway, and skeletal and muscular disorders, thus confirming its association (Figure 4f).

## 3. Discussion

OA incidence and prevalence increases with age [20,24,28,29]. This close relation between aging and OA suggests that aging-related changes provide a basis upon which OA can be initiated [30]. The ability to delay cellular dysfunctions to counteract diseases related to aging initially depends on the understanding of the basic changes that are generated [31].

Senescent cartilage presents different histological characteristics from those observed in young rat cartilage such as decreased toluidine blue staining that suggests a modification of the extracellular matrix, which agrees with the decreases of the proteoglycan content described by age [24,32]. A decrease in Col2a1 expression and an increase in Mmp13 expression as age-related changes in cartilage have been described in other studies [23,26,27]. Some characteristics that suggest an increase in calcification in senescent tissue are an increase in the amount of compact bone in the tissue, an intense and demarcated limiting line, and a regression of the growth plate. It has been postulated that there is an association between the amount of cartilage calcification and OA severity, suggesting that cartilage calcification is a systemically driven process that can have an early onset in life, which could be a causal factor in OA pathogenesis [33]. A decreased number of chondrocytes has been shown for OA, and it is difficult to determine the different layers of the cartilage characteristics that match what have been observed during aging and OA development [20,22,23,24,25]. The primary function of chondrocytes is to maintain cartilage homeostasis, in part through the production of extracellular matrix components [24]. Therefore, a reduction in the number of chondrocytes contributed to the alterations observed in the extracellular matrix in the senescent group, and to increase the Mankin score, which considers alterations present in the structure of the cartilage, cellular abnormalities, and matrix staining, compared to young rat cartilage (Figure 1). 

Autophagy is a strictly catabolic process favoring vital homeostatic pathways for survival during times of metabolic duress [34]. Cell/tissue-specific loss of autophagy has been shown to give rise to neurodegenerative disorders, metabolic defects, and cancers, among other issues [35]. Because it has been suggested that changes generated in the cartilage by age are triggered by a decompensation in the mechanisms that maintain homeostasis, such as autophagy [13,36], we decided to evaluate what happens in this pathway in the cartilage of aged rats.

Our PCR array analysis showed a deregulation of the gene expression of the autophagy pathway between young and senescent rats (Figure 2). A differential expression analysis showed the deregulation of fifteen genes (AKT1, CTSD, Park7, Rps6kb1, Ambra1, Atg16l1, Atg4c, Atg5, Npc1, Esr1, Hdac6, Htt, Map1lc3a, Mapk8, and Tgm2) (Figure 3), considering a fold change of >1.5 and *p*-value of <0.05 as cut-off criteria. Among this group of fifteen genes, all were downregulated (Figure 3b). These genes are important mediators of autophagy associated with chondrocyte aging [9,37]. 

Caramés et al. [36] described that the reduction and loss of expression of the ULK1, beclin1, and Map1lc3a (i.e., LC3) genes can be observed in OA in human and in aging-related and surgically-induced OA in mice, and these results were similar to those observed in our study model. Map1lc3a is a marker of the autophagosome and is structurally and functionally essential for autophagosome formation and cargo recognition [35,38]. Studies have shown that in elderly mice, there is a decreased number of autophagic vesicles (compared to young mice) associated with a reduced expression of autophagy-related 5 (ATG5) and Map1lc3a, and an increase in the apoptotic markers such as poly (ADP-ribose) polymerase 1 [39].

In human chondrocytes, autophagy inhibits the expression of genes associated with cartilage breakdown. The decrease in ATG5 reduces the number of messenger RNA (mRNA) transcripts encoding collagen proteins and type II aggregates, and it increases the number of mRNA transcripts encoding the catabolization of the Mmp13 and ADAM metallopeptidase with thrombospondin type 1 motif 5 (ADAMTS5) enzymes [39].

Other relevant gene is Ambra1, which belongs to the ATG family and is a crucial regulator of autophagy [40]. Ambra1 interacts with Beclin1 through the Vps34/PI3KC3 kinase to assemble a PI3K class III complex, which positively regulates the formation of autophagosomes [41]. For this reason, the decrease in Ambra1 expression in the chondrocyte could be related to the reduction of autophagy. Thus, chondrocytes with OA have a decrease in the proteins involved in the process of autophagy and, at the same time, contribute directly to the pathogenesis of OA. In addition, there is a decreasing order of coregulation among the genes: Htt and Tm9sf1, Ambra1 and Rps6kb1, Npc1 and Ctsd, Hgs and Tgm2, Hdac6 and Park7, and Akt1 and Eif2ak3 (Figure 3a). The confirmation of this co-regulation should be made with other experiments that identify, for example, that these genes are targets of similar transcription factors.

On the other hand, we determined the biological importance of this deregulation by submitting these data to two bioinformatics tools. The analysis with Advaita Bio’s iPathwayGuide confirmed that biological processes involved in this deregulation are related to metabolic mechanisms (Figure 4a). The analysis showed that 11 genes are associated with cellular homeostasis, 6 with autophagosome assembly, and 4 with the aging pathway (Figure 4b,d). 

The in silico analysis performed with the IPA software confirmed that there are different pathways associated with our deregulated genes. Among these, we highlight autophagy, IGF-1 signaling, PI3K/AKT signaling, and mammalian target of rapamycin (mTOR) signaling. All of these pathways contain downregulated genes (Figure 4e). Growth factors like IGF-1 bind to insulin-like growth factor receptors (IGF1Rs), activating AKT which subsequently activates mTOR [42]. It is known that IGF-1 signaling has highly anabolic effects in cartilage, enhances cartilage matrix synthesis, and participate in the regulation of chondrocyte proliferation [43,44,45]. IGF-1 stimulation on the PI3K signaling pathway is responsible for the ability of IGF-I to increase proteoglycan synthesis [46]. Part of the effects of IGF1 are mediated via AKT [47]. Additionally, AKT1 controls cartilage calcification during endochondral ossification and during the formation of osteophytes in OA [48]. IGF-1 has protective effects in chondrocytes stimulated with IL1B, and it is suggested that this is also in part through the regulation of the Src/PI3K/AKT pathway [49]. With aging, there is a decreased response to IGF-1 stimulation in both bone and cartilage [50,51,52,53], as well as an imbalance in Akt and Erk phosphorylation [53]; this is in agreement with our results.

Another related signaling pathway is PTEN signaling. PTEN functions as a negative regulator of both the PI3K/AKT signaling and MEK/ERK signaling pathways, and it is postulated that PTEN regulates matrix synthesis in chondrocytes via the PI3K-AKT signaling pathway under oxidative stress [54]. Since it has been described that the inhibition of AKT by PTEN is required for the maintenance of healthy cartilage, its alteration would favor the appearance of OA [55].

The route of autophagy proposed by IPA includes several genes that are downregulated by age, and part of these genes are also involved in skeletal and muscular disease (14 genes) and the senescence pathway (14 genes) (Figure 4e). For this reason, the impact of this deregulation is not only on the path of autophagy but also on the senescence pathway and skeletal muscle diseases, probably favoring aging of the tissue and the appearance of osteoarthritis and other chronic diseases associated with aging.

OA incidence and prevalence increase with age [20,24,28,29]. It has already been described that autophagy prevents cartilage degeneration from age-related osteoarthritis by facilitating chondrocyte survival [56,57]. Through cell therapy such as platelet-rich plasma, Moussa et al. could reverse the senescence of chondrocytes and they suggest that this effect could be through the promotion of autophagy [58]. Nogueira-Recalde et al. [59] reported that compounds like fibrate partially decrease chondrocyte senescence by increasing autophagic flux.

In such a way, reversing this deregulation could improve the viability of cells in different tissues. In the case of skeletal muscle, aging-mediated anomalous proteostasis could be involved in sarcopenia, and the activation of autophagy could contribute to the maintenance of a healthy cellular environment of skeletal muscle during aging [60]. Other authors have mentioned that an eight-week resistance training could lead to the stimulation of autophagy, a decrease in NLRP3 expression, and a reduction of apoptosis in peripheral blood mononuclear cells in elderly subjects, such that regular physical exercise could be an inducer of autophagy with anti-aging effects [61]. In pathologies such as rheumatoid arthritis, it has been described that the stimulation of autophagy could reduce the apoptosis of chondrocytes [62]. In the case of bone, the induction of autophagy by rapamycin (the pharmacological inhibition of mTOR) may function as an effective therapeutic approach for the promotion of fracture healing [63] and promoting autophagy via the 5’ AMP-activated protein kinase (AMPK)/mTOR pathway might be an effective agent for the prevention of glucocorticoid-induced osteoporosis [64]. Furthermore, autophagy has been described as having a direct role in the intracellular mineralization process of osteoblasts, where it appears to be induced during mineralization [65]. In that article, they mention that the lack of proteins such as Atg5, among others, reduces ex-vivo mineralization capacity. This effect has also been observed during chondrogenesis, where it has been reported that the suppression of autophagy would reduce the growth of proliferative chondrocytes, which would cause a severe growth retardation [66]. 

Autophagy could protect the chondrocyte by inhibiting its aging and the development of OA [67]. Therefore, the identification of genes associated with the autophagy pathway that are deregulated by age could be an interesting alternative to the treatment of this multifactorial and progressive disease. Possible targets of OA treatment that would allow for the creation of new strategies for this chronic disease have been identified. 

The aging phenotype in humans is very heterogeneous and has a time-dependent progressive increase in disease susceptibility [68,69]. These age-related characteristics involve a gradual increase in reactive oxygen species production and genome instability, as well as a progressive decrease in antioxidant, DNA repairing, and proteostatic systems, among others [70]. In fact, it has been reported that in mouse brain tissue, the proteins associated with autophagy do not vary significantly at fairly close ages (young versus adult), but significant differences have been observed between tissues obtained from young and old mice [71]. Despite this, one of the main limitations of our study was not having a group with intermediate ages to understand whether the deregulation profile was maintained during the time or varied in some periods. Because of that, the combination of results obtained from different models with different ages must be considered in order to have a more appropriate understanding of the changes that happened with age [68,69]. Though the intention of this analysis was to demonstrate that an entire pathway was dysregulated in a subset of samples, another limitation of this study was not making a confirmation of this dysregulation through other tools such as protein analysis.

## 4. Materials and Methods 

Rats and tissue collection: All animal experiments were performed according to protocols approved by Scientific Ethics Committee from Universidad de La Frontera, Chile (N° 015/2016). Male Sprague Dawley (SD) rats were purchased from the Faculty of Chemical Sciences of the University of Chile. The rats were housed in a temperature-controlled environment with 12-h light/dark cycles, where they received food and water ad libitum. The animals were separated in two groups: young (*n* = 5) and senescent rats (*n* = 6). Young rats were sacrificed at 3 weeks, and aged rats were sacrificed at 25 month of age. One old rat died as a result of natural causes. We use this model as a model of aging because we were interested in the changes that happened in articular cartilage by age. Knee joints were recollected for analysis. After the sacrifice, the articular cartilage from the tibial plateau and femoral condyles were separated and collected under sterile conditions. The samples used for gene expression were later embedded in RNA for one day and then stored at –80 °C. For the preparation of coronal sections, the knee joints were flexed at 120° and fixed in formaldehyde. Later, the knees from rats were decalcified in 10% EDTA (Merck, Darmstadt, Germany). After that, the knees were embedded in paraffin (Fisher Scientific, Waltham, MA, USA). Sections of 5 μm were collected with 200 μm intervals between every tissue placed in the slide (Superfrost plus) from each paraffin block, according to recommendations of the OARSI histopathology initiative [72]. Then, the slides with paraffin tissue sections were heated at 60 ºC to avoid falling during the staining procedure. 

Histological analysis of rat knees: Sections from paraffin-embedded joint were first deparaffinized, cleared, and hydrated by passing the slides through Xylene (4 min, 3x, Merck, Darmstadt, Germany) and decreasing concentrations of ethanol (100%, 95%, and 70%) for 2 min each before they were finally immersed in distilled water. For the toluidine blue staining, we followed the protocol recommend for OARSI [72]. The slides were mounted with an Entellan mounting medium (Merck, Darmstadt, Germany). Mounted slides were photographed using a Leica S6D 0.63× magnification. The histopathological changes in cartilage were classified using the modified Mankin score system [73]. This score measures the structure of the cartilage, cellular abnormalities, and matrix staining with a total score of 13 points. For the quantification of cells present in the articular cartilage, 5 areas were selected to be photographed for each knee (5 photos per knee), where the upper limit was the surface of the cartilage using the 100× objective in a Leica S6D microscope. Subsequently, cellularity was quantified with the Stepanizer stereology software. Statistically significant differences between two groups were determined with unpaired Student’s t-test. *p* values of less than 0.05 were considered significant. In addition, the immunohistochemistry of Col2a1 (DSHB, #II–II 6B3, 0.4 μg/mL in PBS/1% BSA 1:100) and matrix metalloproteinase 13 (Mmp13, ABCAM, ab39012, 1:200) was performed. After the deparaffinization and rehydration of the tissue sections, Mmp13 and Col2a1, respectively, were immunostained with the two-step immunohistochemistry method instructed by the manufacturer. 

Real Time PCR Array: Total RNA from articular cartilage was isolated with TRIZOL (Invitrogen, Carlsbad, CA, USA) as per the manufacturer’s instructions. The RNA quality and quantity were assessed using a NanoDrop spectrophotometer (Thermo Scientific, Waltham, MA, USA). RNA samples with an A260/280 ratio between 1.8 and 2.0 and an A260/230 ratio between 2.0 and 2.2 were used for RT-qPCR. An equal amount of RNA (1 μm) from each sample was then converted into first-strand cDNA using a RT^2^ First Strand Kit (SABiosciences, Frederick, MD, USA) for a PCR array. The expression of 84 key genes involved in the autophagy pathway was quantified using a PARN-084Z kit from Qiagen and RT^2^ SYBR Green Fluorescein qPCR Master Mix (SABiosciences, Frederick, MD, USA), according to the manufacturers protocol. The expression of each gene was normalized to the best housekeeping genes. We evaluated the reference genes using RefFinder and geNorm, and we found that HRT1 and RPL1 were the best housekeeping genes [74,75]. The results were calculated using the ∆∆CT method. The threshold and baseline values were set manually, and the resulting threshold cycle values (CT) were analyzed using the PCR array data analysis template supplied by the manufacturer’s website (RT^2^ Profiler PCR Array Date Analysis version 3.5. Available online: http://pcrdataanalysis.sabiosciences.com/pcr/arrayanalysis.php (accessed 22 January 2017). *p* values of less than 0.05 were considered significant. 

Predicted functional analysis: Genes that were significantly deregulated among the control and group 1 in the PCR array were selected for analysis through the iPathwayGuide analysis and Ingenuity Pathway Analysis (IPA^®^, Qiagen, Redwood, USA). We applied both software tools to identify significant pathways, cellular processes, and diseases. 

## Abbreviations

ADAMTS5	ADAM metallopeptidase with thrombospondin type 1 motif 5
AKT	Serine/threonine kinase
AMPK	5′ AMP-activated protein kinase
Col2a1	Collagen type II alpha 1 chain
HRT1	Heart protein 1
IGF-1	Insulin-like growth factor 1
Mmp13	Matrix metalloproteinase 13
mRNA	Messenger RNA
mTOR	Mammalian target of rapamycin
OA	Osteoarthritis
OARSI	Osteoarthritis Research Society International
PI3K	Phosphoinositide 3-kinase
RPL1	Ribosomal protein L1
RT-qPCR	Reverse transcription quantitative polymerase chain reaction
SD	Sprague Dawley
ULK1	Unc-51-like autophagy activating kinase 1
Vps34/PI3KC3	Phosphatidylinositol 3-kinase catalytic subunit type 3
